# Spinal Cord Hemisection Facilitates Aromatic L-Amino Acid Decarboxylase Cells to Produce Serotonin in the Subchronic but Not the Chronic Phase

**DOI:** 10.1155/2015/549671

**Published:** 2015-10-04

**Authors:** Bushra Azam, Jacob Wienecke, Dennis Bo Jensen, Aleena Azam, Mengliang Zhang

**Affiliations:** ^1^Department of Neuroscience and Pharmacology, University of Copenhagen, 2200 Copenhagen, Denmark; ^2^Department of Nutrition, Exercise and Sports, University of Copenhagen, 2200 Copenhagen, Denmark; ^3^Department of Experimental Science, Neuronano Research Center, Lund University, 223 81 Lund, Sweden

## Abstract

Neuromodulators, such as serotonin (5-hydroxytryptamine, 5-HT) and noradrenalin, play an essential role in regulating the motor and sensory functions in the spinal cord. We have previously shown that in the rat spinal cord the activity of aromatic L-amino acid decarboxylase (AADC) cells to produce 5-HT from its precursor (5-hydroxytryptophan, 5-HTP) is dramatically increased following complete spinal cord transection. In this study, we investigated whether a partial loss of 5-HT innervation could similarly increase AADC activity. Adult rats with spinal cord hemisected at thoracic level (T11/T12) were used with a postoperation interval at 5 days or 60 days. Using immunohistochemistry, first, we observed a significant reduction in the density of 5-HT-immunoreactive fibers in the spinal cord below the lesion on the injured side for both groups. Second, we found that the AADC cells were similarly expressed on both injured and uninjured sides in both groups. Third, increased production of 5-HT in AADC cells following 5-HTP was seen in 5-day but not in 60-day postinjury group. These results suggest that plastic changes of the 5-HT system might happen primarily in the subchronic phase and for longer period its function could be compensated by plastic changes of other intrinsic and/or supraspinal modulation systems.

## 1. Introduction

Spinal cord injury (SCI) has a devastating effect on daily life of the patients. The foremost and frustrating symptoms are the losses of sensory, motor, and/or autonomic functions [1–5]. Spinal cord injury can be either complete or incomplete, and the symptoms for different SCI individuals may vary according to the severity of the trauma. Neural plasticity occurs over time at sites both above and below the level of injury, which results in both the pathophysiological complications and the functional recovery [6, 7]. The monoaminergic system is one of the systems that undergo drastic plastic changes in the spinal cord following SCI [8–16]. In the mammalian spinal cord monoamine neurotransmitters, for example, serotonin (5-hydroxytryptamine, 5-HT), dopamine, and noradrenaline, are important modulators of both sensory and motor functions. It is commonly believed that monoamines in the spinal cord originate from different supraspinal brain regions [17, 18]. Accordingly in complete SCI the monoamines are largely gone although residual amounts still remain [11, 19], whereas in incomplete SCI monoamines in the spinal cord are lost at a varied degree depending on the severity of the injury [20].

Recently our research group has focused on the plastic changes of serotonergic system in the spinal cord. Using a sacral spinal cord transection rat model we have found that 5-HT2 (A and C) receptors are upregulated in response to complete SCI [13, 14, 16]. More importantly, we have found that cells expressing aromatic L-amino acid decarboxylase (AADC) in the spinal cord, which normally do not contain monoamines [21], increase their activity and could potentially produce 5-HT in the presence of 5-HT precursor, 5-hydroxytryptophan (5-HTP) [22]. Following incomplete SCI, either contusion or hemisection, there remains a considerable amount of 5-HT in the spinal cord below the lesion. For instance, following hemisection the remaining amount of 5-HT below the lesion is about 8–40% of the normal level 3–8 days after the lesion according to the data from different laboratories [9, 10, 23–25]. More importantly some studies have reported a gradual recovery of 5-HT due to reinnervation to the lesion side over time [9, 10, 24] although this is not constantly observed [25, 26]. Then the question is whether this 5-HT is produced from the 5-HT sprouting fibers from the uninjured side or from the intrinsic 5-HT-producing cells, such as AADC cells. This issue is investigated by using a hemisection SCI rat model in the present study. We used rats with a postinjury time at either 5 days or 60 days to examine the expression of 5-HT based on the facts that the descending 5-HT fibers have been degenerated at 5–7 days after the transection [14, 27], that from 4 to 8 weeks after incomplete SCI significant plastic changes have occurred in the spinal cord including the descending cortical and subcortical spinal fibers [6], and that at both time points the AADC cells have shown a steady increased ability to synthesize 5-HT from 5-HTP [22].

## 2. Materials and Methods

### 2.1. Experimental Animals

All experiments followed the guidelines of EU Directive 2010/63/EU and were approved by the Danish Animal Experiments Inspectorate. Efforts were taken to minimize the number of animals and their sufferings. Twenty-nine male Sprague-Dawley rats with initial body weight of 160–490 g during the operation were used in this study. The animals were subjected to either thoracic spinal cord hemisection operation (*n* = 28) or sacral spinal cord transection (*n* = 1). All the rats had a 12/12-hour light/dark cycle and had access to food and water ad libitum. The rats subjected to hemisection were divided into four groups and the rats in each group underwent different treatments before perfusion (see further below).

### 2.2. Spinal Cord Hemisection and Transection Operation

Before the hemisection operation, the rat was anesthetized with 2.0% isoflurane in a mixture of gas of O_2_ (500 psi) and N_2_O (200 psi). The surgical area was shaved and cleaned with alcohol and the whole operation was carried out under sterile conditions. A 0.2 mL mix of sedatives and local anesthesia (Xylocaine 12.5 mg/mL and Marcaine 2.5 mg/mL) were given intramuscularly. Also, a nonsteroidal anti-inflammatory drug and a postoperation pain relieving drug (Rimadyl, 5 mg/kg) were given subcutaneously. The operation was performed under a surgery microscope. For hemisection the laminectomy was done at the thoracic vertebral level T10-T11. The dura was opened and about 1-2 mm of the spinal cord at level T11-T12 was removed at one side without damaging the dorsal vein or ventral artery. The wound was then closed by stitching the muscle, fascia and skin separately with a monofilament suture. For one rat that was subjected to spinal cord transection at the second sacral level (S2) the surgery procedure has been described elsewhere [22]; that is, a small piece of the spinal cord tissue at S2 level was completely removed. After the operation, the rat was subcutaneously treated with analgesic (Temgesic 0.1 mg/kg) at every 8 hours for the first 48 hours. The welfare of the animals was controlled every day until the end of the experiments.

Initially the 28 spinal hemisected rats were divided into two time groups: 16 rats in a 5-day postoperation group and 12 rats in a 60-day postoperation group. In each time group the rats were further divided into two subgroups subjected to different treatments. However, due to compromised welfare of three animals in the 5-day group they had to be euthanized before the planned time. Thus in the end there were 25 rats in total in the four groups: 14 rats were used in experimental groups or treated groups (8 in the 5-day group and 6 in the 60-day group, resp., Table 1) which were subjected to intraperitoneal (i.p.) injection of 5-HTP (50 mg/kg) combined with carbidopa (20 mg/kg) in saline with a small amount of hydrochloride acid 30 min before the perfusion. The other 11 rats were used as control animals or untreated groups (5 in the 5-day group and 6 in the 60-day group, resp., Table 1) and were not injected with any drugs before perfusion. The rat whose spinal cord was transected at S2 level was treated in the same way as those in the experimental groups.

All the rats were euthanized with pentobarbital 50 mg/mL and perfused intracardially with 200–300 mL 0.01 M phosphate buffered saline (PBS) for 3-4 min, followed by a 400 mL solution of 4% paraformaldehyde in 0.1 M phosphate buffer over 15 min. The entire spinal cord was removed and postfixed in the original fixative for 24 hours at 4°C and cryoprotected in PBS with 30% sucrose for 24 hours at 4°C. Upon the removal of the spinal cord the lesion site was inspected under surgery microscope. All the spinal cord hemisected rats used in this study were demonstrated to have been hemisected at T11 or T12 level on one side. The spinal cord was separated into different segments and the lumbar and sacral parts were cut horizontally into 40 *μ*m sections. If the spinal cord was not cut after 48 hours, it was cryoprotected in a 0.01 M PBS solution containing 15% sucrose, 30% ethylene glycol, and 0.05% thimerosal and stored at −20°C.

### 2.3. 5-HT and AADC Immunohistochemistry

Every second section from lumbar (L1–L6) and sacral (S1-Ca3) levels from all the rats was processed for 5-HT and AADC double immunofluorescence staining. The sections were rinsed in PBS twice for 10 min each and in PBS with 0.1% Triton X-100 (PBST) once for 10 min. Then the sections were preincubated in PBST containing 2% bovine serum albumin and 5% normal donkey serum for an hour. After rinsing in PBST for 10 min, the sections were incubated in the same solution containing rabbit anti-5-HT (1 : 10000, Immunostar) and sheep anti-AADC (1 : 200; Millipore-Chemicon) primary antibodies diluted over 24 hours at room temperature. Following rinsing four times in PBST for 15 min each the sections were incubated for an hour in donkey anti-rabbit Alexa Fluor 594 (1 : 200, Invitrogen), donkey anti-sheep Alexa Fluor 488 (1 : 200, Invitrogen) in PBST with 1% bovine serum albumin, and normal donkey serum 2% at room temperature. After rinsing three times in PBS for 10 min each the sections were mounted, dried, and coverslipped with Fluorescence Mounting Medium (Dako).

### 2.4. Data Analysis

The spinal sections were observed with a fluorescence microscope (Leica DM6000B, Leica Microsystems, Wetzlar, Germany). All the images were captured digitally (Leica DFC420 C Digital Camera System) and processed with Adobe Photoshop CS6. To compare with the results from our previous study where the rat spinal cord was transected at S2 level [22] we only analyzed the data acquired from sacrocaudal level in this study.

For the analysis of the density of 5-HT-immunoreactive (IR) fibers, images were taken from 5 or 6 spinal sections. Paired images were taken with an imaging area of 1000 *μ*m × 750 *μ*m from the intermediate gray matter/zone and the ventral motoneuron region from both sides of the selected sections. To avoid bias in the quantitative data analysis due to varied spinal locations images from the injured and uninjured sides were taken from the same rostrocaudal level (mostly at S2–S4 level). The density of 5-HT-IR fibers was analyzed with Image J software [13, 14]. To do this the image was first thresholded and then the area above the threshold level was calculated and the data from injured and uninjured sides were compared. The distribution of AADC cells and the incidence of 5-HT-IR AADC cells at S1-Ca3 were analyzed in the intermediate zone using a MD-plotting system (Accustage) as has been described previously [22]. All the sections containing the AADC cells in the intermediate zone were plotted. The AADC cells and the 5-HT-IR AADC cells were plotted with different symbols. The relative quantity of AADC cells was expressed as cell number per section and the incidence of 5-HT-IR AADC cells was expressed as percent AADC cells for each animal.

Standard deviation (SD) was used to represent data variations from the mean value. The group average value is expressed as the mean ± SD. The statistical analysis was done on Sigmaplot (version 11, Systat Software). Paired *t*-test (or Wilcoxon signed rank test if the data were asymmetrically distributed) or unpaired *t*-test (or Mann-Whitney rank-sum test if the data was asymmetrically distributed) was used for the comparison of data between two groups and the significance level was set at *P* < 0.05.

## 3. Results

### 3.1. Physical Activity of Hind Limbs and Tail after Spinal Hemisection

Immediately after spinal hemisection operation the hind limb on the injured side became paralyzed in all animals. Generally, the rats expressed clumsy locomotion pattern of the affected limb at 5 days and at 7-8 days the locomotor pattern is more functional. After three weeks the affected limb looked, from a qualitative point of view, fully functional during locomotion. We did not score or quantify the level of spasticity of either limb. There was no tail spasticity developed for all the rats regardless of the injury times.

### 3.2. The Density of 5-HT Nerve Fibers in Different Animal Groups

To investigate whether spinal hemisection completely removed 5-HT innervations on the same side and whether the innervations recovered over time we compared the density of 5-HT-IR fibers and 5-HT-IR AADC cells on the injured and the uninjured side at the lumbar and sacrocaudal parts of the spinal cord of the untreated and treated groups at 5 days and 60 days after injury. Since we found a similar labelling pattern of 5-HT-IR fibers and AADC cells at both lumbar and sacrocaudal levels, in this study we mainly focused on the sacrocaudal part in order to compare with our previously published results on completely spinal cord transected rats [22].

**Figure 1:**
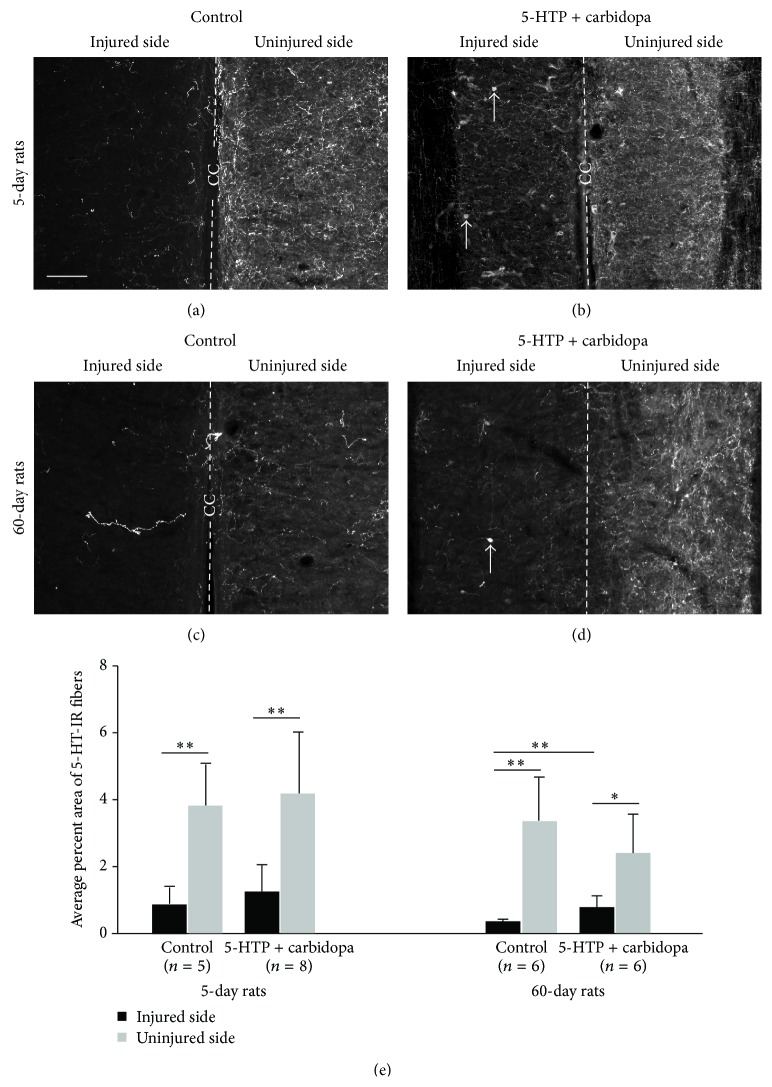
5-HT-immunoreactive (IR) nerve fibers in the intermediate zone in the sacrocaudal spinal cord on the injured and uninjured side in different animal groups. (a–d) Photomicrographs of 5-HT-IR fibers in 5-day untreated group (a) and 5-HTP plus carbidopa treated group (b) and in 60-day untreated group (c) and 5-HTP plus carbidopa treated group (d). Dashed line represents the midline in each horizontal section. CC: central canal. Arrows in (b) and (d) indicate 5-HT-immunoreactive cells. Scale bar in (a), 100 *μ*m. (e) Quantitative data of the density of 5-HT-IR fibers in different animals groups. ^*∗*^ *P* < 0.05, ^*∗∗*^ *P* < 0.01.

First we have analyzed 5-HT fibers in the intermediate zone in 5-day and 60-day groups. In 5-day rats the density of 5-HT-IR fibers in the untreated group was dramatically reduced on the injured side as compared to the uninjured side (Figure 1(a)). When a quantitative analysis was performed in the intermediate zone 5-HT-IR fibers on the injured side were only 23.42% of that on the uninjured side (Table 1). Thus on the uninjured side 5-HT-IR fibers occupied on average 3.80 ± 1.22% of the analyzed area, whereas on the injured side 5-HT-IR fibers occupied only 0.89 ± 0.48% of the analyzed area. This difference was statistically significant (*P* < 0.01, paired *t*-test) (Figure 1(e)). In the treated group, we observed an increase of 5-HT-IR fibers on both the injured and the uninjured side although the 5-HT-IR fibers on the injured side were significantly lower than the uninjured side (Figures 1(b) and 1(e)) (*P* < 0.01, paired *t*-test). Thus, the percent area of 5-HT-IR fibers on the injured side reached 1.26 ± 0.78%, which was 1.4-fold higher than the injured side in the untreated group although no significant difference was reached (unpaired *t*-test). On the uninjured side the 5-HT-IR fiber density was 4.20 ± 1.82%, which was 1.1-fold higher than the uninjured side in the untreated group. Quantitatively in the treated group the density of 5-HT-IR fibers in the injured side was 30.0% (1.26/4.2) of the uninjured side, which was 1.3-fold higher than the untreated group (23.42%). However, for the same side there was no significant difference between treated and untreated groups (unpaired *t*-test) (Figure 1(e)).

Following 60-day injury the density of 5-HT-IR fibers in the untreated group was dramatically reduced on the injured side as compared to the uninjured side (Table 1, Figure 1(c)). The density of 5-HT-IR fibers on the injured side was reduced to 11.11% of that on the uninjured side. Thus on the injured side the density of 5-HT-IR fibers was only 0.36 ± 0.06% on average, whereas on the uninjured side it was 3.24 ± 1.24%. The difference was statistically significant (*P* < 0.01, paired *t*-test) (Figure 1(e)). In the treated group, the density of 5-HT-IR fibers on the injured side was clearly increased (Figure 1(d)), which reached 32.59% of that on the uninjured side although the density on the uninjured side was still significantly higher than the injured side (*P* < 0.05, paired *t*-test) (Figure 1(e)). Thus the density of 5-HT-IR fibers on the injured side was 0.78 ± 0.34%, which was 2.2-fold higher than the injured side in the untreated group and the difference was statistically significant (*P* < 0.01, Mann-Whitney rank-sum test) (Figure 1(e)). On the uninjured side the 5-HT-IR fiber density was 2.41 ± 1.16%, which appeared 41% lower than the uninjured side in the untreated group but the difference was not statistically different (unpaired *t*-test) (Figure 1(e)).

**Figure 2:**
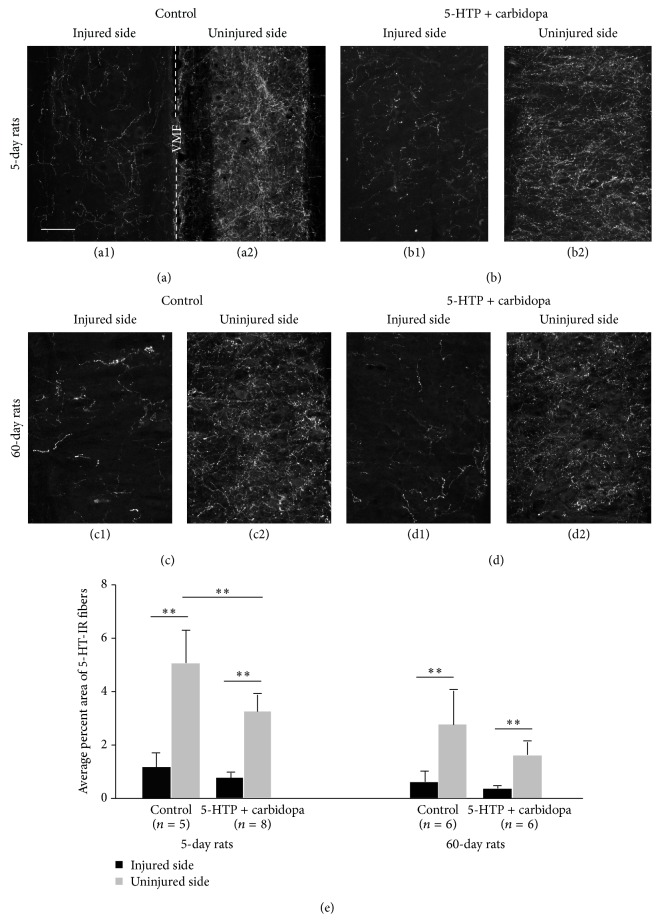
5-HT-immunoreactive (IR) nerve fibers in the ventral horn motoneuron region in the sacrocaudal spinal cord on the injured and uninjured side in different animal groups. (a1–d2) Photomicrographs of 5-HT-IR fibers in 5-day untreated group (a1, a2) and 5-HTP plus carbidopa treated group (b1, b2) and in 60-day untreated group (c1, c2) and 5-HTP plus carbidopa treated group (d1, d2). (a1) and (a2) were from the same horizontal section. Dashed line indicates the midline. VMF: ventral median fissure. Other pairs of photomicrographs were from different sections. Scale bar in (a1), 100 *μ*m. (e) Quantitative data of the density of 5-HT-IR fibers in different animals groups.^*∗∗*^ *P* < 0.01.

Because spinal motoneurons receive dense 5-HT fiber innervations, to investigate whether 5-HT fibers undergo similar plastic changes around the motoneurons, we have examined 5-HT fiber density in different animal groups with the same method. As shown in Table 1 and Figure 2, the density of 5-HT fibers on the injured side was significantly lower than the uninjured side for both 5-day and 60-day groups regardless of the treatment with 5-HTP and carbidopa. Thus the density of 5-HT fibers on the injured side was counted for consistently about 21–24% of that on the uninjured side in the different groups. These density differences on the two sides were somehow similar to those in the intermediate zone (11–33%, see above) in the different groups. However, to our surprise, the density of 5-HT fibers was lower in the treated groups than in the untreated groups for both 5-day and 60-day groups. We will discuss these results further in the Discussion.

We did not try to make a comparison for the 5-HT-IR fiber density between 5-day and 60-day postinjury groups. The key issue we were interested in was that whether 5-HT-IR fibers became denser following 5-HTP + carbidopa application in each group. However, in general the 5-HT-IR density at the 5-day postinjury group was higher than the 60-day postinjury on both sides regardless of locations of the gray matter and the drug applications.

### 3.3. The Distribution of the AADC Cells in Different Experimental Groups

To investigate whether spinal hemisection affects the expression of AADC cells in the spinal cord we investigated the distribution of the AADC cells on the injured and the uninjured side at the sacrocaudal spinal segment in different animal groups. As we have reported previously [22] the AADC cells were found in the dorsal horn, the intermediate zone, and the region around the central canal on both sides (data not shown). To facilitate the comparison with the results from our spinal transection experiments [22] the quantitative analysis was only performed on the data from the intermediate zone.

The results showed that in both 5-day and 60-day groups AADC cells showed a similar distribution on the injured and uninjured side regardless of whether the rat was treated with 5-HTP and carbidopa or not (*P* > 0.05 for the two time groups), indicating that hemisection and drug application did not alter the expression of AADC cells in the injured side (Table 1). Therefore we decided to pool the data from the untreated group and the treated group together. In the 5-day group the number of AADC cells in the intermediate zone on the injured side was 19.60 ± 7.07/section whereas on the uninjured side it was 17.88 ± 8.02/section (*n* = 13). No significant difference was found between the two sides (*P* = 0.721, paired *t*-test). Similarly, in the 60-day group the number of AADC cells in the intermediate zone on the injured side was 10.13 ± 4.82/section whereas on the uninjured side it was 8.98 ± 4.56/section (*n* = 12). No significant difference was found between the two sides (*P* = 0.128, paired *t*-test). Since we did not systematically plot the AADC cells in all the sections no attempt was made to make a quantitative comparison of AADC cells between the 5-day and the 60-day groups.

### 3.4. The Ability of the AADC Cells to Produce 5-HT from Extraneously Applied 5-HTP following Hemisection

Because the main purpose of this study was to investigate whether AADC cells increased their ability to produce 5-HT from 5-HTP following hemisection, we have examined 5-HT expression in AADC cells in two different animal groups following 5-HTP + carbidopa application. 5-HT-positive AADC cells were examined at the sacrocaudal level in both the untreated and the treated group in 5-day and 60-day rats. In addition 5-HT-positive AADC cells were also examined in the spinal cord from the rat subjected to a complete spinal transection at S2 level and the results showed that 100% of the AADC cells in the intermediate zone became 5-HT-IR in this rat (data not shown).

**Figure 3:**
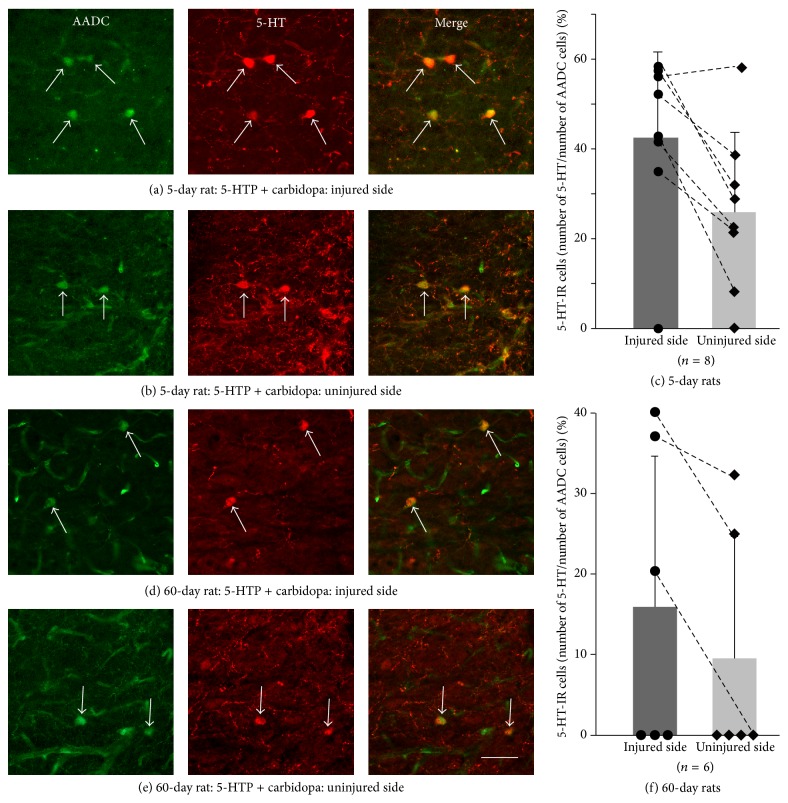
5-HT expression in AADC cells in the intermediate zone in the sacrocaudal spinal cord on the injured and uninjured side in the animal groups injected with 5-HTP and carbidopa. (a) Photomicrograph showing 5-HT-immunoreactive (IR) AADC cells on the injured side in a rat at 5 days after injury. (b) On the uninjured side in a rat at 5 days after injury. Arrows indicate the 5-HT-IR AADC cells. (c) Group data of the incidence of 5-HT-IR AADC cells on the injured side with respect to uninjured side. Dots/squares represent data from different individual cases. Dashed lines connect the data from the same cases. There was significant difference of 5-HT-IR cells between the two sides (^*∗∗*^ *P* < 0.05). (d–f) Data from 60-day postinjury group with the same format as in 5-day postinjury group.

In the untreated groups no AADC cells became 5-HT-positive on both the injured and the uninjured side of the spinal cord in both 5-day and 60-day groups. In the 5-day treated group we have observed a certain number of 5-HT-IR AADC cells on both the injured side and uninjured side in 7 out of 8 cases (Figures 3(a) and 3(b)). Quantitative analysis showed that 42.52 ± 19.15% (*n* = 8) of AADC cells became 5-HT-positive on the injured side whereas only 25.25 ± 17.81% (*n* = 8) of the AADC cells became 5-HT-IR on the uninjured side. The difference was significantly different (*P* < 0.01, power = 0.85 with *α* = 0.05, paired *t*-test) (Figure 3(c)). In 60-day treated group we observed a certain number of 5-HT-IR AADC cells in 3 out of 6 rats in the injured side and 2 out of 6 rats in the uninjured side (Figures 3(d) and 3(e)). Quantitative analysis showed that 15.98 ± 18.72% (*n* = 6) of the AADC cells on the injured side became 5-HT-IR while 9.57 ± 15.00% (*n* = 6) of AADC cells on the uninjured side became 5-HT-IR. The difference between the injured and uninjured sides was not statistically significant (*P* = 0.25, Wilcoxon signed rank test) (Figure 3(f)). Further, we have compared the data in the two time groups treated with 5-HTP and carbidopa. The results revealed that on the injured side the incidence of 5-HT-IR AADC cells in 5-day group was significantly higher than that in 60-day group (*P* < 0.05, power = 0.59 with *α* = 0.05, *t*-test), whereas on the uninjured side the difference was not significant (*P* = 0.096, power = 0.27 with *α* = 0.05, *t*-test).

Due to large variations for the 5-HT-IR AADC cells in different drug treated groups we speculate whether this was related to the variations of 5-HT fiber denervation. Thus we have made a Pearson product-moment correlation analysis between incidences of 5-HT-IR AADC cells and the densities of 5-HT fibers in different drug treated groups and the results showed no correlation between the density of the 5-HT-IR fibers and the number of 5-HT-IR AADC cells. Actually, the variation of the density of 5-HT-IR fibers was relatively small within a specific group/side (Table 1), which indicates that the higher incidences of 5-HT-IR AADC cells in some cases were due to other facts (see Discussion).

## 4. Discussion

In the present study using thoracic hemisection rat model we found that, first, 5-HT-IR nerve fibers in both the intermediate zone and the ventral horn motoneuron region were dramatically reduced on the injured side in comparison with the uninjured side for both 5-day and 60-day groups, and this decrease did not recover in animals with a longer survival time; second, there was no significant expression difference for the AADC cells on the injured side and uninjured side for both time groups; and third, the AADC cells increased their ability to synthesize 5-HT from its precursor, 5-HTP, at 5 days but not at 60 days after injury. These results indicate different plastic changes may occur for the spinal cord which is subjected to a partial or a complete injury.

### 4.1. Plastic Changes of 5-HT Fibers following Partial Spinal Cord Injury

There are many investigations to examine the plastic changes of 5-HT fibers with different SCI animal models. The density of 5-HT fibers in the spinal cord after SCI varies in relation to many different factors which include, among others, the severity of the injury, the postinjury time, and the locations in the spinal cord. For example, when the spinal cord was completely transected usually just a few 5-HT-IR fibers were observed below the lesion in the intermediate zone and/or the ventral horn following more than two months of injury [14, 15, 28]. In contusion injury the density of remaining 5-HT fibers below the lesion depends on the locations along the dorsoventral axis and the severity of the injury [20]. In hemisection the density of 5-HT fibers in the ventral horn was reported at a range from 8% to 30% of the control value according to different studies with a postinjury time window from 4 to 7 days [9, 23, 25, 26]. Hains et al. [10] found that, in the dorsal horn (laminae I-II), following 3-day of hemisection at thoracic level, ~30% 5-HT-IR fibers remain in the lumbar spinal cord. Our 5-HT fiber data from 5-day animals in the intermediate zone and the motoneuron region are in general agreement with that reported in the dorsal horn and the ventral horn, indicating that 5-HT fibers may undergo a similar degradation process in different parts of the gray matter.

An apparent discrepancy exists in the literature as to whether 5-HT fibers recover over time after spinal hemisection. Some studies have reported a gradual increase of 5-HT-IR fibers on the ipsilateral side below the lesion [9, 10, 24] whereas some others did not (e.g., [25]). For example, Saruhashi et al. [9] observed that from first week to the fourth week the 5-HT-IR fibers recovered from 20% to about 75% of the normal value in the ventral horn. Camand et al. [24] and Hains et al. [10] also reported similar results although with slight variations in the intermediate zone and dorsal horn, respectively. On the contrary, Filli et al. [25] reported a decrease to about 10% by 4 weeks from about 30% at day 4. Our results are quite similar to Filli et al.'s in that by 60 days the density of 5-HT-IR fibers in the intermediate zone was reduced to 11% from 23% at 5 days. In the motoneuron region although the value was not reduced so much (23% versus 22%) it neither increased. There might be several explanations for this discrepancy. First, the different locations in the spinal gray matter may undergo different plastic changes of 5-HT innervations. In our study 5-HT fiber density in the ipsilateral intermediate zone decreased more than in the motoneuron region upon 60 days, which may indicate that the intermediate zone is less affected by 5-HT axon reinnervation. Second, upon 5 days after injury the 5-HT fibers may not drop to the lowest level. It may take 2-3 weeks for the degenerated 5-HT fibers to completely disappear [14]. In this case the reinnervation will take some time to reach a certain level. Thus more experimental groups with narrower time intervals are needed. Third, it might be more logical to compare the data from the injured side with the normal control animal rather than the uninjured sides, since hemisection could also affect the 5-HT fiber density in the uninjured side considering the existence of crossing 5-HT fibers. In addition, different lesion level, slight lesion variations, and different analysis methods may also result in different 5-HT density output. In any way, further studies are needed to find out which factor(s) is (are) involved in causing this discrepancy.

We observed that the density of 5-HT-IR fibers in the intermediate zone increased on both sides in 5-day group and on injured side in 60-day group after combining 5-HTP and carbidopa injections, and the density decreased in the motoneuron region for both sides in the two time groups. It is easy to understand the increase due to the increased availability of 5-HT precursor in the 5-HT fibers. However, it is bewildering that 5-HT fiber density decreased with addition of 5-HTP. One possible explanation may be that the contribution of 5-HT fibers from the AADC cells is very limited presumably due to their poor fiber arborization. In this case, what we saw in the motoneuron region is just the results representing approximately those prior to 5-HTP application.

### 4.2. Plasticity of AADC Cells following SCI

Similar to our study using complete spinal transection rat model [22] we did not observe AADC cell expression changes on the injured side. This result is different from Li et al.'s [29] observation that AADC cells are only expressed in the region around the central canal and that only after spinal transection they start to appear in the intermediate zone. The results from our group thus indicate that the number of AADC cells is not increased in response to SCI although AADC plastic changes at molecular level cannot be excluded.

One of the main purposes of the present study is to examine whether AADC cells increase their ability to produce 5-HT from its precursor, 5-HTP. It has been reported both from our and Bennett's research group that after complete spinal transection AADC cells in the spinal cord increase their efficacy to catalyze 5-HTP [22, 29]. In the present study we have observed an increased enzymatic ability in spinal AADC cells on the injured side at 5 days but, to our surprise, not at 60 days although the results are not conclusive due to the lower statistical power in the 60-day group. Thus we have observed a different AADC activity in response to complete spinal transection and hemisection. At 5 days after either complete spinal transection or hemisection AADC cells dramatically increase their ability to use 5-HT precursor to synthesize 5-HT ([22]; present study). This rapid response of AADC cells in the spinal cord may be a response to a sudden loss of supraspinal 5-HT innervation, and thus through the deactivation of 5-HT1B receptors AADC cells are activated [22]. After spinal hemisection both intraspinal and supraspinal plasticity occur which will help eventually to reestablish anatomical and functional circuits in the spinal cord. In addition to the descending 5-HT pathway other descending pathways, such as corticospinal, rubrospinal, and reticulospinal pathway, may also have an inhibitory effect on the spinal AADC cells. A large body of evidence has indicated that extensive plasticity occurs usually after 2-3 weeks of injury (reviewed by Nardone et al. [30]). Indeed, using microdialysis technique Gerin et al. [31] have found that 5-HT release in the lumbar ventral horn showed about 200% increase at day 18 relative to day 8 following subhemisection. Meanwhile plastic changes of many other pathways, including intraspinal and descending pathways, may reorganize and reestablish functional circuits in the spinal cord [6, 32]. All the above factors may contribute to reestablishing an inhibitory network for the AADC cells in the chronic phase following hemisection. In spinal transection these descending pathways, including 5-HT pathway, will not recover below the lesion; thus AADC cells will continue to be active in the chronic phase.

### 4.3. Significance of Plastic Changes of AADC System in the Spinal Cord

5-HT is an important neuromodulator for both motor and sensory functions in the spinal cord [11, 30]. Numerous studies have illustrated that locomotor activity can be recovered following 5-HT or its precursor application or transplantation of 5-HT-producing cells in the spinal cord after spinal cord transection [30, 33–36]. Thus it is extremely important that after SCI there are sources in the spinal cord that could produce 5-HT. Evidence from complete spinal cord transection studies showed that the activity of AADC cells is increased in both the subchronic and chronic phase and 5-HT produced from these cells could induce increased motoneuron excitability and thus muscle spasms. Our results in the present study indicate that after hemisection a plastic change occurs for AADC cells in that in the subchronic phase they could increase their ability to potentially provide 5-HT if its precursor is available. Although AADC cell activity is not significantly increased in chronic phase the cells may help to maintain the reestablished spinal network which is essential for functional recovery of the spinal cord in later phase. Actually in agreement with many other studies (e.g., [9, 25]) we observed a motor function recovery over time in the body parts below the hemisection in 60-day rats without giving any external interventions. It deserves further investigation as to how much AADC cells contribute to this functional recovery.

## Figures and Tables

**Table 1:** Summary of the results from the different animal groups.

Group	Number of animals	Spinal cord side	Density of 5-HT fibers in IMZ^1^ (% of analyzed area)	Density of 5-HT fibers in VH^2^ (% of analyzed area)	Density of AADC cells (number of cells/analyzed section)	Density of 5-HT cells (% of AADC cells)
5-day control	5	Injured	0.89 ± 0.48	1.19 ± 0.51	19.99 ± 3.83	0
		Uninjured	3.80 ± 1.22	5.02 ± 1.21	22.37 ± 7.11	0

5-day 5-HTP + carbidopa	8	Injured	1.26 ± 0.78	0.73 ± 0.18	19.36 ± 8.78	42.52 ± 19.15
		Uninjured	4.20 ± 1.82	3.13 ± 0.61	15.08 ± 7.61	25.86 ± 17.81

60-day control	6	Injured	0.36 ± 0.06	0.59 ± 0.38	8.83 ± 3.45	0
		Uninjured	3.24 ± 1.24	2.72 ± 1.25	7.10 ± 3.73	0

60-day 5-HTP + carbidopa	6	Injured	0.78 ± 0.34	0.35 ± 0.08	11.42 ± 5.94	15.98 ± 18.72
		Uninjured	2.41 ± 1.16	1.54 ± 0.46	10.85 ± 4.80	9.57 ± 15.00

^1^IMZ: intermediate zone; ^2^VH: ventral horn.
